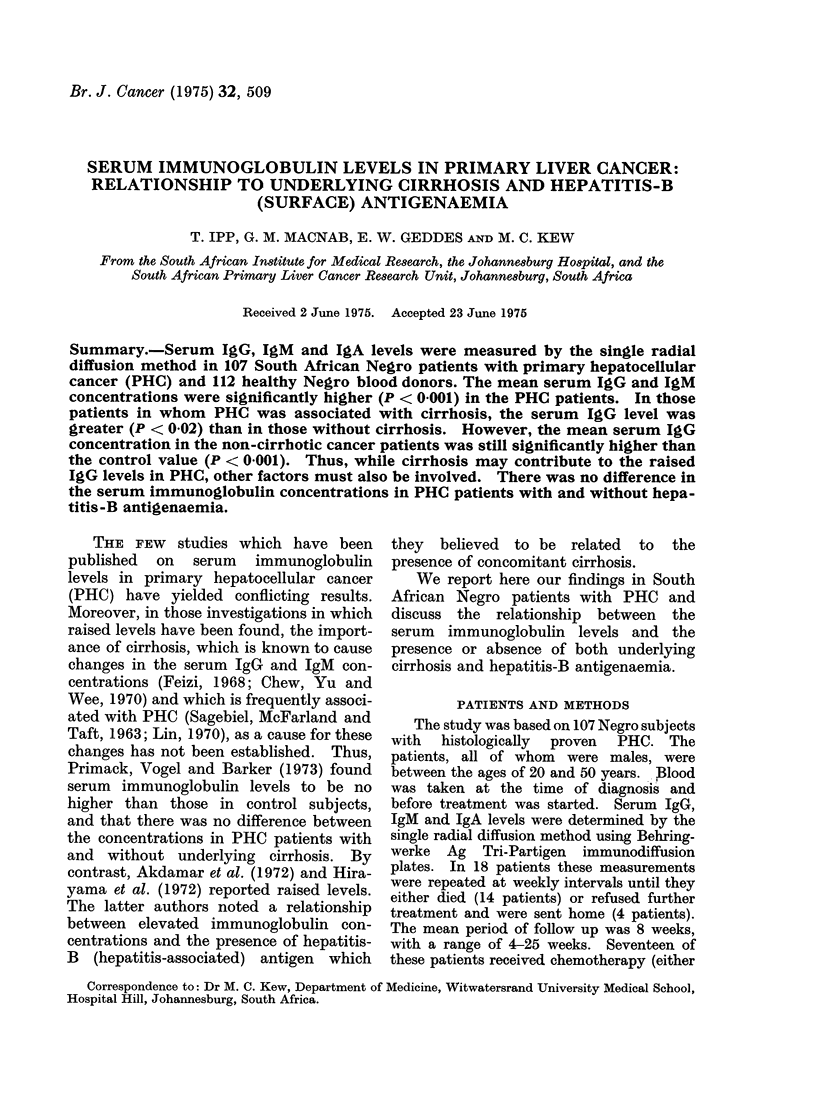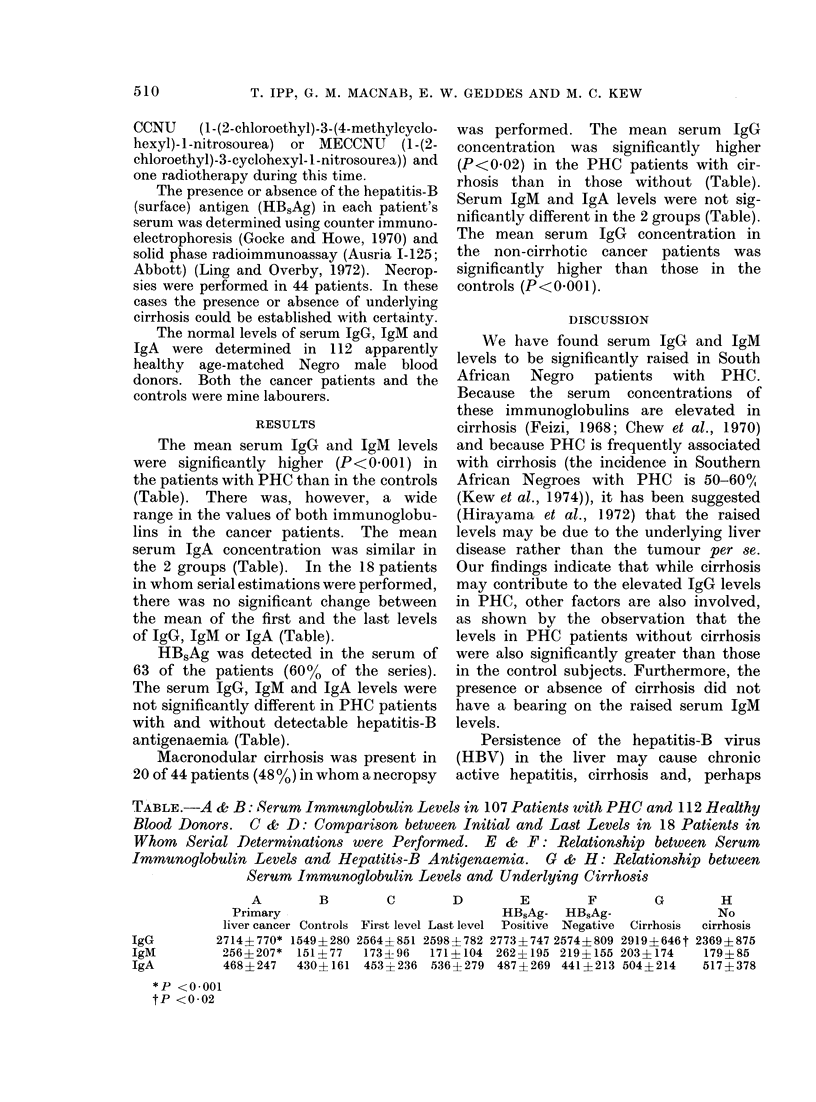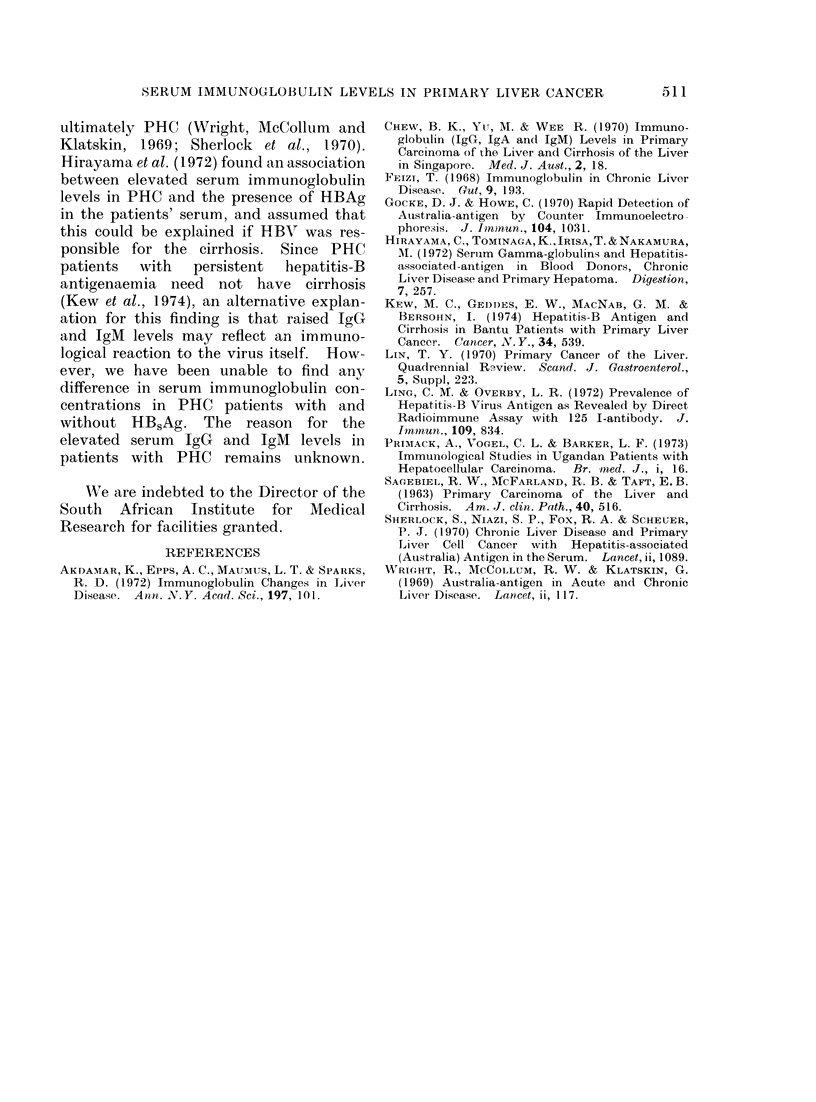# Serum immunoglobulin levels in primary liver cancer: relationship to underlying cirrhosis and hepatitis-B (surface) antigenaemia.

**DOI:** 10.1038/bjc.1975.253

**Published:** 1975-10

**Authors:** T. Ipp, G. M. Macnab, E. W. Geddes, M. C. Kew

## Abstract

Serum IgG, IgM and IgA levels were measured by the single radial diffusion method in 107 South African Negro patients with primary hepatocellular cancer (PHC) and 112 healthy Negro blood donors. The mean serum IgG ANd IgM concentrations were significantly higher (P less than 0-001) in the PHC patients. In those patients in whom PHC was associated with cirrhosis, the serum IgG level was greater (P less than 0-02) than in those without cirrhosis. However, the mean serum IgG concentration in the non-cirrhotic cancer patients was still significantly higher than the control value (P less than 0-001). Thus, while cirrhosis may contribute to the raised IgG levels in PHC, other factors must also be involved. There was no difference in the serum immunoglobulin concentrations in PHC patients with and without hepatitis-B antigenaemia.


*Br. J. Cancer* (1975) **32**, 509

### SERUM IMMUNOGLOBULIN LEVELS IN PRIMARY LIVER CANCER: RELATIONSHIP TO UNDERLYING CIRRHOSIS AND HEPATITIS-B (SURFACE) ANTIGENAEMIA

T. IPP, G. M. MACNAB, E. W. GEDDES AND M. C. KEW

*From the South African Institute for Medical Research, the Johannesburg Hospital, and the South African Primary Liver Cancer Research Unit, Johannesburg, South Africa*

Received 2 June 1975. Accepted 23 June 1975

**Summary.—Serum IgG, IgM and IgA levels were measured by the single radial diffusion method in 107 South African Negro patients with primary hepatocellular cancer (PHC) and 112 healthy Negro blood donors. The mean serum IgG and IgM concentrations were significantly higher ($P < 0{\cdot}001$) in the PHC patients. In those patients in whom PHC was associated with cirrhosis, the serum IgG level was greater ($P < 0{\cdot}02$) than in those without cirrhosis. However, the mean serum IgG concentration in the non-cirrhotic cancer patients was still significantly higher than the control value ($P < 0{\cdot}001$). Thus, while cirrhosis may contribute to the raised IgG levels in PHC, other factors must also be involved. There was no difference in the serum immunoglobulin concentrations in PHC patients with and without hepatitis-B antigenaemia.**

THE FEW studies which have been published on serum immunoglobulin levels in primary hepatocellular cancer (PHC) have yielded conflicting results. Moreover, in those investigations in which raised levels have been found, the importance of cirrhosis, which is known to cause changes in the serum IgG and IgM concentrations (Feizi, 1968; Chew, Yu and Wee, 1970) and which is frequently associated with PHC (Sagebiel, McFarland and Taft, 1963; Lin, 1970), as a cause for these changes has not been established. Thus, Primack, Vogel and Barker (1973) found serum immunoglobulin levels to be no higher than those in control subjects, and that there was no difference between the concentrations in PHC patients with and without underlying cirrhosis. By contrast, Akdamar *et al.* (1972) and Hirayama *et al.* (1972) reported raised levels. The latter authors noted a relationship between elevated immunoglobulin concentrations and the presence of hepatitis-B (hepatitis-associated) antigen which they believed to be related to the presence of concomitant cirrhosis.

We report here our findings in South African Negro patients with PHC and discuss the relationship between the serum immunoglobulin levels and the presence or absence of both underlying cirrhosis and hepatitis-B antigenaemia.

### PATIENTS AND METHODS

The study was based on 107 Negro subjects with histologically proven PHC. The patients, all of whom were males, were between the ages of 20 and 50 years. Blood was taken at the time of diagnosis and before treatment was started. Serum IgG, IgM and IgA levels were determined by the single radial diffusion method using Behringwerke Ag Tri-Partigen immunodiffusion plates. In 18 patients these measurements were repeated at weekly intervals until they either died (14 patients) or refused further treatment and were sent home (4 patients). The mean period of follow up was 8 weeks, with a range of 4–25 weeks. Seventeen of these patients received chemotherapy (either

Correspondence to: Dr M. C. Kew, Department of Medicine, Witwatersrand University Medical School, Hospital Hill, Johannesburg, South Africa.

CCNU (1-(2-chloroethyl)-3-(4-methylcyclohexyl)-1-nitrosourea) or MECCNU (1-(2-chloroethyl)-3-cyclohexyl-1-nitrosourea)) and one radiotherapy during this time.

The presence or absence of the hepatitis-B (surface) antigen ($HB_sAg$) in each patient's serum was determined using counter immunoelectrophoresis (Gocke and Howe, 1970) and solid phase radioimmunoassay (Ausria I-125; Abbott) (Ling and Overby, 1972). Necropsies were performed in 44 patients. In these cases the presence or absence of underlying cirrhosis could be established with certainty.

The normal levels of serum IgG, IgM and IgA were determined in 112 apparently healthy age-matched Negro male blood donors. Both the cancer patients and the controls were mine labourers.

### RESULTS

The mean serum IgG and IgM levels were significantly higher ($P < 0 \cdot 001$) in the patients with PHC than in the controls (Table). There was, however, a wide range in the values of both immunoglobulins in the cancer patients. The mean serum IgA concentration was similar in the 2 groups (Table). In the 18 patients in whom serial estimations were performed, there was no significant change between the mean of the first and the last levels of IgG, IgM or IgA (Table).

$HB_sAg$ was detected in the serum of 63 of the patients (60% of the series). The serum IgG, IgM and IgA levels were not significantly different in PHC patients with and without detectable hepatitis-B antigenaemia (Table).

Macronodular cirrhosis was present in 20 of 44 patients (48%) in whom a necropsy was performed. The mean serum IgG concentration was significantly higher ($P < 0 \cdot 02$) in the PHC patients with cirrhosis than in those without (Table). Serum IgM and IgA levels were not significantly different in the 2 groups (Table). The mean serum IgG concentration in the non-cirrhotic cancer patients was significantly higher than those in the controls ($P < 0 \cdot 001$).

### DISCUSSION

We have found serum IgG and IgM levels to be significantly raised in South African Negro patients with PHC. Because the serum concentrations of these immunoglobulins are elevated in cirrhosis (Feizi, 1968; Chew *et al.*, 1970) and because PHC is frequently associated with cirrhosis (the incidence in Southern African Negroes with PHC is 50–60% (Kew *et al.*, 1974)), it has been suggested (Hirayama *et al.*, 1972) that the raised levels may be due to the underlying liver disease rather than the tumour *per se*. Our findings indicate that while cirrhosis may contribute to the elevated IgG levels in PHC, other factors are also involved, as shown by the observation that the levels in PHC patients without cirrhosis were also significantly greater than those in the control subjects. Furthermore, the presence or absence of cirrhosis did not have a bearing on the raised serum IgM levels.

Persistence of the hepatitis-B virus (HBV) in the liver may cause chronic active hepatitis, cirrhosis and, perhaps

TABLE.—*A & B: Serum Immunglobulin Levels in* 107 *Patients with PHC and* 112 *Healthy Blood Donors. C & D: Comparison between Initial and Last Levels in* 18 *Patients in Whom Serial Determinations were Performed. E & F: Relationship between Serum Immunoglobulin Levels and Hepatitis-B Antigenaemia. G & H: Relationship between Serum Immunoglobulin Levels and Underlying Cirrhosis*

| | A | B | C | D | E | F | G | H |
|---|---|---|---|---|---|---|---|---|
| | Primary liver cancer | Controls | First level | Last level | $HB_sAg$-Positive | $HB_sAg$-Negative | Cirrhosis | No cirrhosis |
| IgG | 2714±770* | 1549±280 | 2564±851 | 2598±782 | 2773±747 | 2574±809 | 2919±646† | 2369±875 |
| IgM | 256±207* | 151±77 | 173±96 | 171±104 | 262±195 | 219±155 | 203±174 | 179±85 |
| IgA | 468±247 | 430±161 | 453±236 | 536±279 | 487±269 | 441±213 | 504±214 | 517±378 |

* $P < 0 \cdot 001$
† $P < 0 \cdot 02$

ultimately PHC (Wright, McCollum and Klatskin, 1969; Sherlock *et al.*, 1970). Hirayama *et al.* (1972) found an association between elevated serum immunoglobulin levels in PHC and the presence of HBAg in the patients' serum, and assumed that this could be explained if HBV was responsible for the cirrhosis. Since PHC patients with persistent hepatitis-B antigenaemia need not have cirrhosis (Kew *et al.*, 1974), an alternative explanation for this finding is that raised IgG and IgM levels may reflect an immunological reaction to the virus itself. However, we have been unable to find any difference in serum immunoglobulin concentrations in PHC patients with and without $HB_SAg$. The reason for the elevated serum IgG and IgM levels in patients with PHC remains unknown.

We are indebted to the Director of the South African Institute for Medical Research for facilities granted.

### REFERENCES

AKDAMAR, K., EPPS, A. C., MAUMUS, L. T. & SPARKS, R. D. (1972) Immunoglobulin Changes in Liver Disease. *Ann. N.Y. Acad. Sci.*, **197**, 101.

CHEW, B. K., YU, M. & WEE R. (1970) Immunoglobulin (IgG, IgA and IgM) Levels in Primary Carcinoma of the Liver and Cirrhosis of the Liver in Singapore. *Med. J. Aust.*, **2**, 18.

FEIZI, T. (1968) Immunoglobulin in Chronic Liver Disease. *Gut*, **9**, 193.

GOCKE, D. J. & HOWE, C. (1970) Rapid Detection of Australia-antigen by Counter Immunoelectrophoresis. *J. Immun.*, **104**, 1031.

HIRAYAMA, C., TOMINAGA, K., IRISA, T. & NAKAMURA, M. (1972) Serum Gamma-globulins and Hepatitis-associated-antigen in Blood Donors, Chronic Liver Disease and Primary Hepatoma. *Digestion*, **7**, 257.

KEW, M. C., GEDDES, E. W., MACNAB, G. M. & BERSOHN, I. (1974) Hepatitis-B Antigen and Cirrhosis in Bantu Patients with Primary Liver Cancer. *Cancer, N.Y.*, **34**, 539.

LIN, T. Y. (1970) Primary Cancer of the Liver. Quadrennial Review. *Scand. J. Gastroenterol.*, **5**, Suppl, 223.

LING, C. M. & OVERBY, L. R. (1972) Prevalence of Hepatitis-B Virus Antigen as Revealed by Direct Radioimmune Assay with 125 I-antibody. *J. Immun.*, **109**, 834.

PRIMACK, A., VOGEL, C. L. & BARKER, L. F. (1973) Immunological Studies in Ugandan Patients with Hepatocellular Carcinoma. *Br. med. J.*, i, 16.

SAGEBIEL, R. W., MCFARLAND, R. B. & TAFT, E. B. (1963) Primary Carcinoma of the Liver and Cirrhosis. *Am. J. clin. Path.*, **40**, 516.

SHERLOCK, S., NIAZI, S. P., FOX, R. A. & SCHEUER, P. J. (1970) Chronic Liver Disease and Primary Liver Cell Cancer with Hepatitis-associated (Australia) Antigen in the Serum. *Lancet*, ii, 1089.

WRIGHT, R., MCCOLLUM, R. W. & KLATSKIN, G. (1969) Australia-antigen in Acute and Chronic Liver Disease. *Lancet*, ii, 117.